# Role of the lectin pathway of complement in hematopoietic stem cell transplantation-associated endothelial injury and thrombotic microangiopathy

**DOI:** 10.1186/s40164-021-00249-8

**Published:** 2021-12-19

**Authors:** Eleni Gavriilaki, Vincent T. Ho, Wilhelm Schwaeble, Thomas Dudler, Mohamed Daha, Teizo Fujita, Sonata Jodele

**Affiliations:** 1 Hematology Department-BMT Unit, G Papanikolaou Hospital, Leof. Papanikolaou, Pilea Chortiatis 570 10, Thessaloniki, Greece; 2 Department of Medical Oncology, Dana-Farber Cancer Institute, 450 Brookline Ave, Boston, MA 02215 USA; 3 Department of Veterinary Medicine, University of Cambridge, Cambridge, CB3 0ES UK; 4 Discovery and Development, Omeros Corporation, 201 Elliott Ave W, Seattle, WA 98119 USA; 5 Department of Nephrology, Leiden University Medical Center, Albinusdreef 2, 2333 ZA Leiden, Netherlands; 6 Department Fukushima Prefectural General Hygiene Institute, 61-Watari-Nakakado, Fukushima, Fukushima 960-8141 Japan; 7 Division of Bone Marrow Transplantation and Immune Deficiency, Cincinnati Children’s Hospital Medical Center, 3333 Burnet Ave, Cincinnati, OH 45229 USA

**Keywords:** Endothelial injury, Complement activation, Lectin pathway, Hematopoietic stem cell transplantation-associated thrombotic microangiopathy

## Abstract

Hematopoietic stem cell transplantation-associated thrombotic microangiopathy (HSCT-TMA) is a life-threatening syndrome that occurs in adult and pediatric patients after hematopoietic stem cell transplantation. Nonspecific symptoms, heterogeneity within study populations, and variability among current diagnostic criteria contribute to misdiagnosis and underdiagnosis of this syndrome. Hematopoietic stem cell transplantation and associated risk factors precipitate endothelial injury, leading to HSCT-TMA and other endothelial injury syndromes such as hepatic veno-occlusive disease/sinusoidal obstruction syndrome, idiopathic pneumonia syndrome, diffuse alveolar hemorrhage, capillary leak syndrome, and graft-versus-host disease. Endothelial injury can trigger activation of the complement system, promoting inflammation and the development of endothelial injury syndromes, ultimately leading to organ damage and failure. In particular, the lectin pathway of complement is activated by damage-associated molecular patterns (DAMPs) on the surface of injured endothelial cells. Pattern-recognition molecules such as mannose-binding lectin (MBL), collectins, and ficolins—collectively termed lectins—bind to DAMPs on injured host cells, forming activation complexes with MBL-associated serine proteases 1, 2, and 3 (MASP-1, MASP-2, and MASP-3). Activation of the lectin pathway may also trigger the coagulation cascade via MASP-2 cleavage of prothrombin to thrombin. Together, activation of complement and the coagulation cascade lead to a procoagulant state that may result in development of HSCT-TMA. Several complement inhibitors targeting various complement pathways are in clinical trials for the treatment of HSCT-TMA. In this article, we review the role of the complement system in HSCT-TMA pathogenesis, with a focus on the lectin pathway.

## Background

Over the last 50 years, hematopoietic stem cell transplantation (HSCT) has evolved into the standard of care for patients with otherwise fatal hematologic, metabolic, and neoplastic disorders [1, 2]. However, physical, chemical, and immunologic stressors during the transplantation process (conditioning regimens, radiotherapy, chemotherapy, immunosuppressive drugs, cytokines released during the engraftment process, and allogeneic reactions of donor-derived immune cells) perturb endothelial cells, precipitating endothelial injury [3]. Microvascular endothelial injury following HSCT places patients at risk for long-term organ damage and death [3].

Endothelial injury has been shown to be centrally involved in the pathophysiology of several HSCT-associated conditions [2, 4], collectively termed endothelial injury syndromes (EIS). EIS include hepatic veno-occlusive disease/sinusoidal obstruction syndrome, idiopathic pneumonia syndrome, diffuse alveolar hemorrhage, capillary leak syndrome, graft-versus-host disease (GVHD), and thrombotic microangiopathy (TMA) [2, 4]. These conditions are not discrete diseases, but different clinical manifestations stemming from endothelial injury. Thus, these syndromes often overlap in presentation and classification [2, 5]. In HSCT-associated TMA (HSCT-TMA), endothelial injury commonly affects the kidneys as well as the gastrointestinal tract, lungs, heart, central nervous system, and, rarely, the retina [3, 6]. These manifestations typically occur during the first 6 months after HSCT [7, 8] and reflect sites of underlying tissue damage [2].

Endothelial injury triggers activation of the complement system—significantly through the lectin pathway—via altered cell-surface patterns on injured endothelial cells, initiating an inflammatory response [7]. Activation of the lectin pathway further triggers the coagulation cascade [9]. Both complement activation and the coagulation cascade lead to a procoagulant state that reduces vascular integrity, promotes platelet adhesion, increases vasodilation, and promotes leukocyte infiltration [3]. Subsequent thrombus formation and tissue injury can lead to organ-specific damage, multiorgan dysfunction, or death [10].

Here we discuss evidence for the role of lectin pathway activation in endothelial injury-associated complications of HSCT and how targeting complement activity may provide therapeutic benefit for patients with HSCT-TMA.

### Endothelial injury syndromes and clinical manifestations

#### Normal function of endothelial cells

Endothelial cells fulfill essential homeostatic functions within the circulatory system, as they form the contact interface between blood and the perfused tissues and organs [10]. Normally, a balance of procoagulant and anticoagulant pathways is poised to react to injury and to attenuate any imbalance before tissue damage [10]. Endothelial cells control these pathways by maintaining vascular tone and platelet activation, and by regulating prothrombotic and thrombolytic events [10]. In addition, endothelial cells mediate immune functions: healthy endothelium is responsible for diapedesis of leukocytes into injured and inflamed tissues and platelet-leukocyte interactions [10, 11].

#### Endothelial injury

In damaged endothelium, blood components such as C-reactive protein (CRP) enter the interstitial space and induce inflammation [10]. Impaired endothelial functions increase risk of disease by abrogating normal immune response, vascular tone, and transport of electrolytes and fluid [2]. Persistent activation of endothelium can lead to a procoagulant state, increasing the risk of stasis and endothelial injury [3, 12]. The resulting injury may cause organ damage and thrombosis, which can lead to arteriosclerosis, peripheral vascular disease, stroke, and hypertension [10]. Risk of endothelial injury is increased by diabetes, obesity, hypertension, and environmental factors such as smoking [10, 13, 14].

Endothelial injury is common in HSCT. Conditioning regimens (including chemotherapy and/or radiation) prior to transplantation, agents given for prevention of acute GVHD such as calcineurin or mammalian target of rapamycin (mTOR) inhibitors, infection, and the graft-versus-host reaction itself can all contribute to endothelial injury [3, 15]. The site and severity of endothelial injury associated with HSCT determine the presentation and classification of the syndrome (Table 1) [2].

**Table 1:** Hematopoietic stem cell transplantation-related endothelial injury syndromes

Syndrome	Incidence	Diagnostic criteria	Clinical presentation	Clinical outcomes
Veno-occlusive disease (VOD)/sinusoidal obstruction syndrome (SOS)	2–60% [16, 17, 152, 153]	Triad of weight gain (often ascites), right upper quadrant pain or hepatomegaly, and elevated bilirubin [16, 17]	Fluid retention and ascites, jaundice, weight gain (≥ 5%), and painful hepatomegaly, in the absence of other identifiable causes of liver disease. Platelet transfusion refractoriness is an early sign, particularly in pediatric patients. Pediatric onset can present beyond 30 days after HSCT [17, 152]	100-day mortality (all causes): [17, 154] Mild VOD/SOS: 3% Moderate VOD/SOS: 20% Severe VOD/SOS: > 80%
Idiopathic pneumonia syndrome (IPS)/ diffuse alveolar hemorrhage (DAH)	2–14% [19]	Signs and symptoms of diffuse or multilobar pneumonia, with evidence of alveolar injury, after infectious causes have been ruled out by bronchoscopy or lung biopsy [19]	**IPS:** Shortness of breath, cough, hypoxemia, with or without fever. May progress rapidly to ARDS/hypoxemic respiratory failure requiring intubation. Onset usually within 120 days after HSCT [19, 155] **DAH:** Fever, dyspnea, and hypoxemia; occurs within 30 days after HSCT and within 5 days of neutrophil engraftment [19]	Mortality: [18] Overall: 60–86% Patients requiring mechanical ventilation: > 95%
Engraftment syndrome (ES)	13% [157]	Major: noninfectious fever, erythroderma > 25% of body surface, noncardiogenic pulmonary edema Minor: hepatic dysfunction, renal insufficiency, weight gain, transient encephalopathy [3, 156]	Fever, generalized rash, shortness of breath. Usually transient and improves quickly with steroids [156]	Mortality: 18% [156]
Capillary leak syndrome (CLS)/ fluid overload	CLS: 5% [22] Overall fluid overload: 43–66%; Grade ≥ 2 fluid overload: 6–21% [5]	Weight gain, general edematous syndrome that does not respond to furosemide treatment [3]	**CLS:** Pulmonary edema, total body volume overload with weight gain, but relative intravascular volume depletion [156] **Fluid overload:** Fluid retention, weight gain [5]	Mortality: [156] CLS in combination with other EIS: 60% 100-day nonrelapse mortality: [5] Overall fluid overload: 5–9% Grade ≥ 2 fluid overload: 30–36%
Graft-versus-host disease (GVHD)	40–80% Grade II to IV GVHD [158]	**Chronic GVHD: **Diagnosis based on either the presence of specific diagnostic signs or distinctive signs in at least one target organ (skin and appendages, mouth, eyes, genitalia, esophagus, lungs, muscles, and fascia) and by additional confirmation (e.g., biopsy or other objective diagnostic test) [159]	**Acute GVHD:** Skin: inflammatory maculopapular erythematous skin rash Liver: elevated bilirubin GI tract: anorexia with weight loss, nausea, vomiting, diarrhea, severe pain, GI bleeding and/or ileus **Chronic GVHD **may involve: skin, nails, scalp and body hair, mouth, eyes, esophagus, lungs, muscles, joints and fascia, genitalia [159]	Overall mortality: [12] Acute GVHD: 16% Grade IV GVHD: 92%
Hematopoietic stem cell transplantation-associated thrombotic microangiopathy (HSCT-TMA)	Adults: 4–68% [8, 30–36] Pediatrics: 3–39% [24, 27–29]	Overlapping criteria from diagnostic algorithms: schistocytosis, increase in serum LDH levels, thrombocytopenia, anemia, or negative Coombs test Additional criteria: proteinuria, hypertension, terminal complement assay results [3]	Hemolytic anemia with evidence of microangiopathy. Acute renal dysfunction, proteinuria, uncontrolled hypertension. Neurologic dysfunction, encephalopathy, seizures. May also involve lungs leading to pulmonary vascular hypertension, respiratory failure. Intestinal TMA leads to intestinal ischemia, pain, and lower GI bleeding [3, 8, 29]	Non-relapse mortality: [29, 160] Moderate TMA: 34–44% Severe TMA: > 90%

*ARDS* Acute respiratory distress syndrome, *CLS* Capillary leak syndrome, *DAH* Diffuse alveolar hemorrhage, *ES* Engraftment syndrome, *GI* Gastrointestinal, *GVHD* Graft-versus-host disease, *HSCT* Hematopoietic stem cell transplantation, *HSCT-TMA* Hematopoietic stem cell transplantation-associated thrombotic microangiopathy, *IPS* Idiopathic pneumonia syndrome, *LDH* Lactate dehydrogenase, *TMA* Thrombotic microangiopathy, *VOD/SOS* Veno-occlusive disease/sinusoidal obstruction syndrome

#### Clinical manifestations of endothelial injury

We present EIS as different clinical manifestations stemming from the common underlying pathophysiology of endothelial injury, rather than as distinct diseases. Damage due to endothelial injury may occur throughout the body, with overlap in target sites due to similar characteristics across EIS [2]. The reported incidence of EIS in HSCT recipients varies widely due to differences among current diagnostic criteria and heterogeneity within study populations, especially in adults [12].

In some EIS, platelets adhere to endothelium and aggregate to form microthrombi [15]. These thrombi can cause thrombocytopenia and intravascular hemolysis due to red cell fragmentation, leading to tissue hypoxemia and organ damage and failure [15]. Hepatic veno-occlusive disease (VOD)/sinusoidal obstruction syndrome (SOS) occurs when sinusoidal endothelial injury from the conditioning regimen leads to hepatic central vein or sinusoidal thrombosis [16]. Extravasation of blood cells and release of cellular debris into the space of Disse further result in extraluminal compression of sinusoidal vessels, causing sinusoidal obstruction, portal hypertension, sodium-avid fluid retention, ascites, painful hepatomegaly from hepatic capsular distention, and jaundice [17]. Ultimately, multiorgan dysfunction and death can occur [16, 17].

Another of the organ-specific EIS, idiopathic pulmonary syndrome (IPS), is an umbrella term to describe any noninfectious disorder of the lungs characterized by multifocal acute lung injury, shortness of breath, cough, and hypoxemia that occurs within the first four months after HSCT [18]. The pathophysiology of IPS is not completely understood [12], and responses to high doses of corticosteroids are suboptimal [19]. Tumor necrosis factor-α (TNF-α) may play a role in the pathogenesis of IPS and the TNF inhibitor etanercept has been evaluated for management of IPS in combination with corticosteroids, although results were not definitively conclusive [20]. A subset of patients with IPS develop diffuse alveolar hemorrhage (DAH), a form of pulmonary injury that occurs at the time of engraftment, wherein diagnosis is established by the appearance of increasing bloody returns on serial bronchiolar lavage fluids [19]. DAH pathology is thought to be associated with damage to pulmonary arterioles, capillaries, venules, and the alveolar-capillary basement membrane [3].

EIS with systemic presentations include engraftment syndrome (ES), capillary leak syndrome (CLS), and fluid overload. ES occurs at the time of neutrophil engraftment after HSCT, and is believed to involve the unbridled release of proinflammatory cytokines, degranulation products, and activation of complement, leading to systemic endothelial injury [21]. ES usually manifests as fevers, shortness of breath from pulmonary vascular leak, and transient rash that generally respond well to a short course of corticosteroids [21]. CLS is characterized by the loss of intravascular fluids into interstitial spaces due to endothelial injury [22]. In fluid overload, fluid retention and weight gain may require ongoing diuretic therapy or be associated with organ dysfunction [5].

GVHD is the most commonly expected complication of allogeneic HSCT [23]. Acute GVHD is mediated by donor T lymphocytes targeting host tissue, causing unspecified vascular inflammation and endothelial injury early after HSCT, while chronic GVHD tends to occur later and is mediated by a complex interplay between donor effector and regulatory T cells, B cells, and tissue macrophages [2, 23]. Both acute and chronic GVHD can establish conditions that increase the risk of HSCT-TMA, while the presence of HSCT-TMA increases the risk of GVHD [2, 3, 24]. Endothelial injury may represent a common link between GVHD and HSCT-TMA [25]: it is considered the common denominator for both conditions, but excessive complement activation distinguishes HSCT-TMA from GVHD [26].

HSCT-TMA, also known as transplant-associated thrombotic microangiopathy (TA-TMA), occurs when endothelial injury and microthrombus formation cause microangiopathic hemolytic anemia, thrombocytopenia, and organ damage [3]. Reported incidence rates for HSCT-TMA range from 3 to 39% in children [24, 27–29], and from 4 to 68% in adults [8, 30–36]. Differing awareness of HSCT-TMA and screening practices across institutions may reflect differences in identifying the condition, rather than variable incidence in the population. In addition, these reported rates may underestimate the incidence of HSCT-TMA given the nonspecific symptoms, variability among current diagnostic criteria, and heterogeneity within study populations, which have contributed to misdiagnosis and underdiagnosis of the disorder [37–41].

**Fig. 1:**
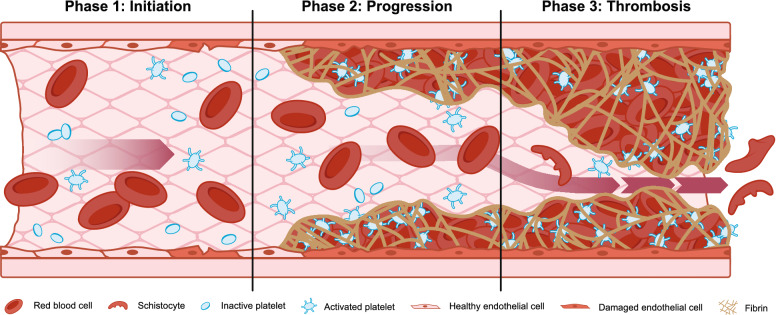
Role of the complement system, including the lectin pathway, in pathophysiology of HSCT-TMA [7, 15]. In *Phase 1 (Initiation)*, factors associated with hematopoietic stem cell transplantation such as calcineurin and mTOR inhibitors, acute graft-versus-host disease, infection, or total body irradiation lead to endothelial injury. In *Phase 2 (Progression)*, the lectin pathway of complement is activated and complement proteins cause further endothelial injury, leading to platelet aggregation and microthrombi formation. In *Phase 3 (Thrombosis)*, further microthrombi formation and mechanical damage lead to HSCT-TMA, organ damage, and organ failure. *HSCT-TMA* hematopoietic stem cell transplantation-associated thrombotic microangiopathy, *mTOR* mammalian target of rapamycin

The pathophysiology of HSCT-TMA is believed to occur in three phases (Fig. 1). The initiation phase of HSCT-TMA pathogenesis results from endothelial injury caused by immunosuppressive agents such as calcineurin or mTOR inhibitors, acute GVHD, infection, or conditioning with cytotoxic agents and/or total body irradiation [15]. During the progression phase, complement activation including the lectin pathway, which is activated by altered carbohydrate and acetylated ligand patterns on injured endothelial cells, causes additional damage, particularly in the microvasculature [7, 15]. In the thrombosis phase, platelets aggregate and form microthrombi, causing consumptive thrombocytopenia, hemolysis from red cell fragmentation, and organ damage [15].

### Endothelial injury activates the complement system

**Fig. 2:**
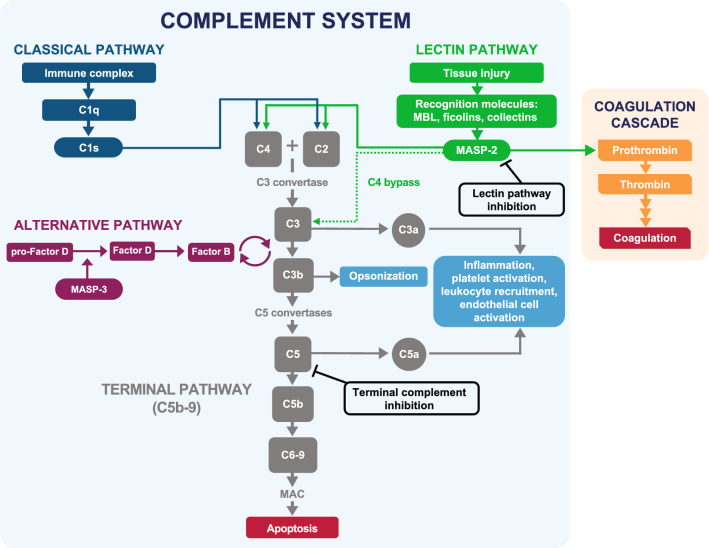
Complement activation pathways [42, 48, 52]. The three complement activation pathways—the classical, lectin, and alternative pathways—eliminate or clear infection or damaged host cells. The *classical pathway* initiates complement activation through antibody binding to immune complexes. The *lectin pathway* is initiated when pattern-recognition molecules bind to certain molecular patterns presented on damaged, malignant or distressed self-tissue or on microbes. The *alternative pathway* acts as an amplification loop of the classical or lectin pathways. All three pathways converge to mediate cleavage of C3, leading to initiation of the terminal pathway and assembly of the MAC. The *coagulation cascade* can be activated via MASP-2 cleavage of prothrombin to thrombin and cleavage of Factor XII to XIIa. *MAC* membrane attack complex, *MASP* mannan-binding lectin-associated serine proteases, *MBL* mannose-binding lectin

A cascade of cellular distress signals can trigger complement activation, leading to a plethora of biologic events: activation of the cellular immune system, chemotactic direction of immune cells to sites of injury, proinflammatory stimulation of leukocytes, promotion of cell‒cell interactions, and the generation of lytic or sublytic membrane attack complexes (MACs) that lyse or mark targeted cells [42, 43]. In a balanced system, the three complement activation pathways—the classical, lectin, and alternative pathways (Fig. 2)—eliminate or clear infection or damaged host cells [42, 43]. When the complement system is dysregulated, excessive complement activation results in damage [44].

#### Complement pathways

The classical pathway initiates complement activation through antibody binding to immune complexes: the globular heads of C1q bind to the Fc portion of IgM or to clusters of IgG fixed to antigens [43]. C1q may also identify foreign surfaces containing proteoglycan patterns (e.g., chondroitin sulfate [serglycin]) [43]. When C1q binds a target, the C1q-associated protease C1r is activated through a conformational change to cleave its only substrate C1s, which in turn cleaves and activates complement proteins C4 and C2 [43]. The resultant complex C4b2b (formerly C4b2a) is known as C3 convertase [45]. The classical pathway was thought to be the predominant pathway activated by endothelial injury [46, 47], but subsequent studies have elucidated that endothelial injury primarily activates the lectin pathway of complement [48–51].

Activation of the lectin pathway, a scavenger system, is initiated when pattern-recognition molecules (PRMs) bind to certain molecular patterns presented on damaged, malignant or distressed self-tissue, or ligands on microbes [43, 52]. Specific molecular patterns exposed on the surface of necrotic, apoptotic, distressed or otherwise injured host cells are termed damage-associated molecular patterns (DAMPs) and those on microbes are termed pathogen-associated molecular patterns [43, 52]. PRMs may also bind fragments and debris of viruses. In HSCT, highly cytotoxic treatment leaves behind many injured endothelial cells that trigger lectin pathway activation.

The lectin pathway-activating carbohydrate pattern-binding class of PRMs known as lectins include mannose-binding lectin (MBL), collectins (CL-10 and CL-11), and ficolins (ficolin-1, ficolin-2, and ficolin-3), which bind to specific ligands on bacteria, viruses, and injured cells [53]. Lectin pathway-specific proteases known as MBL-associated serine proteases 1, 2, and 3 (MASP-1, MASP-2, and MASP-3) form activation complexes with the collectins and ficolins [53]. Juxtaposition of discrete PRM/MASP activation complexes initiates lectin pathway activation [54]; binding of these activation complexes to DAMPs in close proximity to each other facilitates the conversion of MASPs from their zymogen form into their enzymatically active form [53, 55–57]. Cleavage of C4 and C2 via MASP-2 results in formation of C3 convertase [53, 55–57]. Notably, MASP-2 is the only MASP that can cleave both C2 and C4; hence, in the absence of MASP-2, the lectin pathway cannot generate C3 convertase [58–61].

The alternative pathway balances a low-grade steady state of activation with the ability to respond to damage or infection [43, 52]. Factor D cleaves Factor B associated with C3b or C3(H_2_O), generating a C3 convertase [C3bBb or C3(H_2_O)Bb] [62, 63]. MASP-3 was recently shown to be essential for alternative pathway functional activity, as it is required to convert pro-Factor D into its enzymatically active form [63–66]. Hence, in the absence of MASP-3 functional activity, the alternative pathway is deficient [63, 65, 66]. The alternative pathway primarily acts as an amplification loop of the classical or lectin pathways, triggered by formation of C3b [59, 64–66]. Spontaneous hydrolysis of C3 to C3(H_2_O) allows for continuous turnover of C3 and generation of C3 convertase to initiate the alternative pathway [43]. Crosstalk between different pathways of complement supports a rapid response to triggers, and tight regulation prevents collateral damage [60, 67]. A single study observed that properdin may act as a PRM and bind DAMPs on injured host cells, initiating complement activation via the alternative pathway [68]. However, no follow-up studies have been published that corroborate a functional role of properdin as a PRM.

All three pathways converge to mediate the cleavage of C3 into C3a and C3b. C3a, along with C5a, is a potent anaphylatoxin with proinflammatory, prothrombotic, and chemotactic functions that trigger leukocyte recruitment and cytokine production [42, 53]. Accumulation of C3b on the cell surface leads to opsonization of debris and bacteria for clearance [42]. Binding of C3b to C3 convertase forms C5 convertase (C4b2b3b from the classical/lectin pathway, or C3bBb3b from the alternative pathway), which cleaves C5 into C5a and C5b, initiating the terminal pathway [42]. C5b recruits C6, C7, C8, and multiple C9s, resulting in formation of the MAC [42]. The MAC promotes further endothelial damage and may result in apoptosis [69].

Induction of a procoagulant state can be initiated not only by C3a and C5a but also via MASP-2 cleavage of prothrombin to thrombin; MASP-2 can activate the coagulation cascade via activation of prothrombin [70] and by the cleavage of Factor XII to XIIa [71], and activation of the cascade drives fibrin deposition and clot formation [9, 72]. The coagulation cascade [70]—in concert with endothelial damage arising from complement activation—leads to thrombosis, which can result in stroke, hypertension, and peripheral vascular disease [10].

#### Endothelial injury triggers lectin pathway activation in related diseases

As the lectin pathway of complement is activated in response to endothelial injury, evidence for complement activation has been demonstrated in ischemia of various organs, including the kidney and heart [48, 73, 74]. Direct activation of the lectin pathway was identified in animal models of ischemia/reperfusion associated with skeletal muscle, intestinal, myocardial, and kidney injuries [51]. In certain instances of kidney ischemia, the alternative pathway amplifies cleavage of C3 after initiation through the lectin pathway [51, 75]. Activation of the lectin pathway is triggered by local CL-11 upregulation in post-ischemic kidney tissue [76], and CL-11–driven lectin pathway activation may play a central role in tubulointerstitial injury broadly associated with proteinuric renal diseases [77, 78]. Lectin pathway activity also occurs during progression of ischemic brain damage [79–83], with the lectin pathway recognition molecules MBL [84, 85] and ficolin-3 [82] identified as independent predictors of ischemic stroke outcome. Furthermore, MASP-2 was found to play a crucial role in ischemia/reperfusion of a murine model: MASP-2‒deficient mice were protected against myocardial and gastrointestinal injury arising from ischemia/reperfusion [58]. In a later study, use of an anti–MASP-2 antibody in a murine model conferred cardioprotection against myocardial infarction [86].

Rapidly developing research in severe acute respiratory syndrome coronavirus 2 (SARS-CoV-2) has demonstrated the role of endothelial injury in COVID-19. Endothelial injury in patients with COVID-19 activates the complement system, leading to thrombosis and acute respiratory distress syndrome (ARDS) [87]. Microvascular deposits of C5b-9, C4d, and MASP-2 in patients with COVID-19 suggest the role of the lectin pathway in proinflammatory sequelae [87]. Lectin pathway PRMs such as MBL, ficolin-2, and CL-11 bind to spike and nucleocapsid (N) proteins of SARS-CoV-2, contributing to subsequent lectin pathway-mediated deposition of C3b and C4b [88]. Moreover, N proteins within the coronavirus family, including SARS-CoV, MERS-CoV, and SARS-CoV-2, are bound to MASP-2 through an evolutionarily conserved motif [88, 89]. This interaction results in hyperactivation of the complement system and suggests that the lectin pathway is a promising target for coronavirus-induced pneumonia [89]. In small case series, anti-complement therapies have been associated with COVID-19 improvements [89–93]. The C5 inhibitor eculizumab resulted in recovery and reduced mean CRP levels in four patients with COVID-19 [92], although the global Phase 3 clinical trial evaluating the longer-acting C5 inhibitor ravulizumab was recently discontinued for lack of efficacy [94]. The C5a inhibitor BDB-001 has shown promising results in patients with severe COVID-19 [95]. The C3 inhibitor AMY-101 resulted in successful treatment of a patient with severe ARDS due to COVID-19 pneumonia [91] and is being investigated for the management of ARDS caused by COVID-19 in a Phase 2 placebo-controlled trial [96], although a Phase 1/2 trial of the C3 inhibitor APL-9, a pegylated form of AMY-101, for the treatment of severe COVID-19 was recently discontinued due to lack of efficacy [97]. The MASP-2 inhibitor narsoplimab was associated with rapid and sustained reduction of circulating endothelial cell counts and serum IL-6, IL-8, CRP, and lactate dehydrogenase (LDH) in six patients with COVID-19 on mechanical ventilation, correlating with clinical improvement [93].

Collectively, these data suggest a key role for complement, and particularly the lectin pathway, in the pathogenesis of diseases associated with endothelial injury.

### Complement activation underlies the pathology and diagnosis of HSCT-TMA

Complement activation plays an essential role in the pathology of EIS, including HSCT-TMA. The “Three-Hit Hypothesis” (Table 2) outlines sequential risks that facilitate development and progression of HSCT-TMA [7, 15, 98]. The first “hit” toward HSCT-TMA comprises inherent or nonmodifiable risk factors, such as underlying predisposition to complement activation via genetic risk factors or prior endothelial injury [15]. The second “hit” involves transplant-associated risk factors, such as cytotoxic conditioning regimens during HSCT that cause endothelial injury [7, 15]. The third “hit” toward development of HSCT-TMA includes post-transplant risk factors (medications, GVHD, infection, and/or circulating antibodies) that may initiate complement activation [7, 15].

**Table 2:** The “Three-Hit Hypothesis” for development of hematopoietic stem cell transplantation-associated thrombotic microangiopathy (HSCT-TMA) [7, 15]

Inherent/ non-modifiable risk factors	Transplant-associated risk factors	Post-transplant risk factors
Underlying predispositions: Female sex African American ethnicity Severe aplastic anemia CMV seropositive recipient Prior stem cell transplant Genetic variants	Endothelial injury and procoagulant endothelium: Transplant conditioning Total-body irradiation Unrelated donor transplants HLA mismatch Other factors	Post-HSCT initiators of complement activation: Calcineurin inhibitors mTOR inhibitors aGVHD Infection

Adapted with permission from [7]

Sequential risks facilitate development and progression of HSCT-TMA. *The first “hit”* comprises inherent or nonmodifiable risk factors, such as underlying predisposition to complement activation via genetic risk factors. The *second “hit”* involves transplant-associated risk factors such as cytotoxic conditioning regimens that cause endothelial injury. The *third “hit”* includes post-transplant risk factors that may initiate complement activation, such as medications, aGVHD, infection, and/or circulating antibodies

*aGVHD *Acute graft-versus-host disease,* CMV *Cytomegalovirus, *HLA *Human leukocyte antigen, *HSCT* Hematopoietic stem cell transplantation, *mTOR *Mammalian target of rapamycin

Evidence for the role of complement in HSCT-TMA can be found in both pediatric and adult populations. Elevated serum levels of the terminal complement complex C5b-9 are observed in both children and adults with HSCT-TMA [24, 29, 99–102]. In addition, a higher level of complement activation is detected in sera or plasma from patients with HSCT-TMA compared with patients without TMA after HSCT [26, 103].

#### Genetic evidence indicates a role for complement in HSCT-TMA

Genetic abnormalities in complement system proteins and regulatory components are associated with increased complement activity and risk of TMA in HSCT recipients [102, 104]. Recent genetic analysis demonstrated that adult patients with HSCT-TMA possessed significantly more pathogenic, rare variants in regulatory and coding regions of *ADAMTS13, C3, CFB, CFH, CFI, CD46, CFHR3, CD55,* and *THBD* than non-TMA HSCT control recipients [102]. These variants were detected in approximately three fifths of patients who did not respond to conventional treatment and approximately two thirds of patients who died from transplant-associated complications [102].

Genetic variations have also been detected in pediatric HSCT-TMA populations. In an analysis of 17 candidate genes known to participate in complement activation, variations were identified in 65% of patients with HSCT-TMA versus 9% of non-TMA HSCT controls [104]. Incidence of HSCT-TMA and number of gene variants were both higher in nonwhite versus white HSCT recipients [104]. In a different study, transcriptome analyses collected before HSCT, at onset of HSCT-TMA, and after resolution of HSCT-TMA in children showed upregulation of all three complement pathways during active HSCT-TMA that then returned to normal levels after treatment with eculizumab [105].

#### Diagnostic and prognostic markers in HSCT-TMA

Pediatric diagnostic criteria for HSCT-TMA are relatively well established [29]; however, diagnostic criteria in adults are less clear due to the lack of robust natural history studies [37–41]. Variability across current guidelines for HSCT-TMA diagnosis is shown in Table 3, demonstrating the need for universally accepted diagnostic criteria for HSCT-TMA in adults. Moreover, uniform standards for diagnosis and prognosis of HSCT-TMA may expand understanding of markers for disease onset and progression [29] and would be important for clinical management [3].

**Table 3:** Nonspecific diagnostic criteria for HSCT-TMA

Parameter	Blood and Marrow Transplant Clinical Trials Network (2005) [37]	International Working Group (2007) [38]	Overall TMA grouping (2010) [39]	City of Hope (2013) [40]	American Society of Hematology–European Society for Blood and Marrow Transplantation (2014) [41]	Jodele criteria (2014) [29] *Pediatric*
Schistocytes	√	√	√	√	√	√
Elevated LDH	√	√	√	√	√	√
Thrombocytopenia		√	√	√	√	√
Decreased hemoglobin		√	√		√	√
Negative Coombs test	√		√		√	
Increased serum creatinine	√			√		
Decreased haptoglobin		√	√			
Elevated soluble C5b-9					√	√
Proteinuria					√	√
Hypertension					√	√
Other	Neurologic dysfunction				TA-TMA Index ≥ 20	

*LDH* Lactate dehydrogenase, *HSCT-TMA* Hematopoietic stem cell transplantation-associated thrombotic microangiopathy, *TA-TMA* Transplant-associated thrombotic microangiopathy, *TMA* Thrombotic microangiopathy

√ = presence of parameter in HSCT-TMA diagnostic criteria

Tissue histology remains the gold standard in HSCT-TMA diagnosis, but the inherent risk involved with biopsy for HSCT recipients has led clinicians to seek other less-invasive diagnostic markers [106]. Based on results of pediatric studies, the earliest indications of endothelial injury can occur 10 to 14 days before HSCT-TMA diagnosis [29]. In pediatric populations, hypertension, proteinuria, and elevated serum LDH levels have emerged as early signals of HSCT-TMA [29, 30]. Grade II to IV acute GVHD is also an independent risk factor for pediatric HSCT-TMA [30].

Increased markers of endothelial and complement activation correlate with TMA and other EIS after HSCT. Immunohistochemistry of complement has been used to diagnose and characterize HSCT-TMA [26, 106–108]. Deposition of C5b-9 and C4d have been observed in blood vessels and organs of patients with HSCT-TMA [106, 108], and these patients have higher levels of soluble C5b-9 and endothelial activation markers (e.g., thrombomodulin) compared with patients without HSCT-related complications [26, 29]. Another method to identify patients with increased complement activity is the modified Ham test, which has been used to show a higher level of complement activation in patients with HSCT-TMA versus control recipients of HSCT [26, 103, 109]. Finally, significantly higher levels of MASP-2 have been reported in patients with TMAs after allogeneic HSCT [110].

Suppressor of tumorigenicity 2 (ST2) is another molecular marker under consideration for diagnosis of HSCT-TMA [25]. When measured 14 days after HSCT, elevated ST2 is associated with an increased risk of HSCT-TMA in pediatric and young-adult recipients of HSCT [25]. ST2 is also associated with treatment resistance and death in patients with GVHD [111, 112]. In adults, preliminary evidence from the Mount Sinai Acute GVHD International Consortium (MAGIC) suggests that a combined test for ST2 and regenerating islet-derived 3α (REG3α) may predict development of HSCT-TMA [113]. When measurement of these two biomarkers from blood samples taken one week after allogeneic HSCT was used to determine risk category (high vs low), HSCT-TMA developed in seven of 18 high-risk patients and 13 of 88 low-risk patients (p = 0.041) [113]. Other diagnostic tests in development include measuring levels of thrombomodulin, calpain, and haptoglobin degradation product [7].

A few prognostic markers have been identified in pediatric populations, but further characterization is necessary in adult recipients of HSCT. Initial approaches to characterize early symptom patterns in both adults and children during HSCT-TMA may be associated with better treatment outcomes [108]. In pediatric patients predicted to have a moderate risk of HSCT-TMA, elevated soluble C5b-9 levels were associated with higher risk of mortality than was nephrotic range proteinuria [24]. Expression of ST2, a possible diagnostic marker of HSCT-TMA as previously discussed, before HSCT was also found to be a prognostic marker for one-year nonrelapse mortality and severe GVHD [112].

The Endothelial Activation and Stress Index (EASIX)—a composite measure of LDH, creatinine, and platelet levels—has demonstrated prognostic value for risk of death in patients with GVHD [114–116]. In adult patients with GVHD and HSCT-TMA, soluble C5b-9 levels were strongly associated with EASIX score 100 days after transplant and at last follow-up [115]. When EASIX was calculated before conditioning, the score was a significant prognostic factor for HSCT-TMA and was predictive of overall survival after HSCT, independent of other assessments [114]. EASIX score before conditioning also correlated with biomarkers of endothelial homeostasis (e.g., CXCL8/IL-18 and free IL-18) [114].

Established risk factors for mortality associated with HSCT-TMA reflect the multifactorial nature of this condition [15, 33]. In pediatric recipients of HSCT, proteinuria and plasma levels of soluble C5b-9 are negatively associated with survival: 1-year survival rates are significantly lower in patients with elevated plasma C5b-9 levels or proteinuria 30 mg/dL or higher [29]. Furthermore, the antecedent conditions of HSCT-TMA affect survival rates: patients with idiopathic or drug-related HSCT-TMA have longer survival after diagnosis than patients with other precipitating events [33]. Additional risk factors for mortality may include anemia (hemoglobin below 9 g/dL), liver dysfunction, and gastrointestinal bleeding [30]. Intestinal TMA following HSCT emerges as a distinct condition from HSCT-TMA that results in higher mortality rates [117] and is an unfavorable predictor of overall survival by multivariate analysis (p = 0.048) [118].

### Treatment of HSCT-TMA

#### Standard of care

The standard of care for HSCT-TMA aims to resolve the physiologic stress that leads to complement activation and endothelial injury. For patients who do not meet the high-risk criteria for HSCT-TMA, recommended management strategies include reducing or withdrawing calcineurin inhibitor treatment (following risk–benefit assessment) and providing supportive care [7, 108]. For patients with high-risk HSCT-TMA, these measures do not markedly improve survival: one study reported similar rates of hematologic resolution in patients withdrawn from calcineurin inhibitor treatment versus those who continued calcineurin inhibitor treatment (28% vs 29%, respectively) [33]. Hazard ratios for death were not appreciably different between the two groups even after adjusting for covariates [33].

Therapeutic plasma exchange (TPE) historically has been used as an urgent treatment for TMAs, including HSCT-TMA [119, 120]. In complement-mediated TMAs, TPE is thought to remove activated complement components and replenish complement regulators [120]. Early initiation of TPE in ten patients with HSCT-TMA unresponsive to conventional care was associated with laboratory resolution of microangiopathy in nine patients and improved kidney function and HSCT-TMA survival in five patients, suggesting that early TPE may be beneficial in selected patients [119]. However, many patients with HSCT-TMA do not respond to TPE, and the long-term results of TPE in complement-mediated TMAs are poor [7, 121].

Defibrotide is approved by the FDA and EMA for treatment of VOD/SOS [122, 123] and due to its endothelial protective properties, several retrospective studies and case series have investigated defibrotide for the treatment of HSCT-TMA. Outcomes have been varied: an early retrospective study reported death of all five pediatric HSCT-TMA patients treated with defibrotide [124], while another study reported response to defibrotide in four of five children and two of seven adults with HSCT-TMA [125]. In a large retrospective study of 539 HSCT recipients, 64 of whom developed HSCT-TMA, defibrotide treatment was associated with a favorable outcome based on univariate analysis (p = 0.02) [126]. More recent retrospective surveys have found response rates to defibrotide ranging from 65 to 77% in adult and pediatric patients with HSCT-TMA [127, 128]. In a case series, three adults with HSCT-TMA responded to treatment with low-dose defibrotide (< 10 mg/kg/day), although one later died of sepsis [129].

#### Targeting the complement system

Reducing or inhibiting complement activity has shown promise in selected patients with HSCT-TMA during clinical trials (Table 4). Several therapeutics under clinical investigation target the terminal pathway by inhibiting C5, while one targets the lectin pathway via inhibition of MASP-2. These potential treatments are discussed in further detail below. Additional complement inhibitors that have been approved or under development for other complement-mediated diseases [130] may also be relevant in HSCT-TMA, but their clinical utility in this syndrome is currently not known.

**Table 4:** Complement inhibitors under clinical investigation for treatment of HSCT-TMA

Drug	Target/mechanism of action	Class	Company	Status	ClinicalTrials.gov
Eculizumab	C5 inhibition	mAb	Alexion Pharmaceuticals	Phase 2 ongoing; off-label use in clinic [161]	NCT03518203 (pediatric + adult) [162]
Ravulizumab (ALXN1210)	C5 inhibition	mAb	Alexion Pharmaceuticals	Phase 3 ongoing	NCT04543591 (adolescent + adult) [141] NCT04557735 (pediatric) [142]
Nomacopan (Coversin)	C5 and LTB4 inhibition	Recombinant protein	Akari Therapeutics	Phase 3 ongoing	NCT04784455 (pediatric) [145]
Narsoplimab (OMS721)	MASP-2 inhibition	mAb	Omeros Corporation	Phase 2 complete	NCT02222545 (adult) [147]

*HSCT-TMA* Hematopoietic stem cell transplantation-associated thrombotic microangiopathy, *LTB4* Leukotriene B4, *mAb* Monoclonal antibody, *MASP-2* Mannan-binding lectin-associated serine protease 2

The monoclonal antibody eculizumab binds C5 to prevent cleavage into C5a and C5b, inhibiting generation of C5a (one of two primary complement anaphylatoxins) and terminal complement activity [131]. Eculizumab has been studied in small trials and case series with mixed outcomes for adult and pediatric patients [24, 101, 132–136]. Data supporting treatment of adult patients with HSCT-TMA are limited; in two case reports, adult patients with HSCT-TMA had hematologic and/or renal responses to eculizumab treatment [133, 134]. A retrospective analysis of nine adults and three children treated with eculizumab for TMA showed a hematologic response in six patients, including four adults [135]. In a different case series, initial hematologic responses to eculizumab were observed, but long-term overall survival was poor in adult patients [136]. Despite limitations of availability and early initiation, complement inhibition seems to offer improved survival compared to best available treatment so far. Better patient selection might help to identify patients who are in need of a complement inhibitor or patients who might be resistant to eculizumab [102, 137].

Among pediatric populations, the largest eculizumab trial to date established a diagnostic protocol to identify patients with high-risk HSCT-TMA, which has the poorest prognosis [24, 138]. Across pediatric patients (N = 64), 64% had measurable responses, 56% achieved complete remission, and there were no HSCT-TMA relapses during the study [24]. Patients with high-risk HSCT-TMA and elevated complement activation showed poorer outcomes after eculizumab treatment (odds ratio = 0.15, p = 0.0014) and received more doses of eculizumab (*r* = 0.43, p = 0.0004) [24]. Taken together, these results indicate that early initiation of eculizumab treatment and adjustment of dosing seems to be beneficial in this pediatric subpopulation. Since patients with HSCT-TMA suffer from multiple comorbidities, long-term effects of eculizumab require further investigation.

Ravulizumab (ALXN1210) is a C5 inhibitor engineered from eculizumab that possesses a longer terminal half-life, allowing for extended dosing intervals [139]. Like eculizumab, ravulizumab binds C5 with high affinity [140]. In a Phase 3 trial for atypical hemolytic uremic syndrome, ravulizumab treatment resulted in complete TMA response in 54% of patients with no unexpected adverse events [139]. Phase 3 trials for ravulizumab in both adult and pediatric populations with HSCT-TMA are currently ongoing [141, 142].

Nomacopan (formerly Coversin) is a bifunctional inhibitor of C5 and leukotriene B4 that blocks terminal complement activity [143]. In a case report of pediatric HSCT-TMA resistant to eculizumab (due to a *C5* variant), nomacopan showed promising results. Daily treatment with nomacopan improved LDH levels and reticulocyte count and decreased classical pathway hemolytic assay (CH50) below the lower limit of normal [144]. A two-part Phase 3 trial evaluating dosing and efficacy of nomacopan for pediatric HSCT-TMA is ongoing [145].

Narsoplimab (OMS721) is a fully human immunoglobulin gamma 4 (IgG4) monoclonal antibody that binds MASP-2, the effector enzyme of the lectin pathway, and thereby blocks lectin-mediated complement activation [110]. Targeting the lectin pathway without affecting the function of the classical pathway maintains the body’s ability to adopt adaptive immune defense mechanisms for protection against encapsulated organisms, such as *Neisseria meningitis* [146]. Narsoplimab was studied in adult patients with severe HSCT-TMA (N = 28) in a single-arm, open-label trial [147]. Overall, 61% of patients (17/28) who received at least one dose of narsoplimab achieved a response based on improvement in laboratory markers and organ function, and 74% of patients (17/23) who received per-protocol narsoplimab (at least four doses) responded to treatment [148]. One-hundred–day survival post-HSCT-TMA diagnosis was 68% among all patients, 83% among the per-protocol population, and 94% among responders [148]. The most commonly reported adverse events were fever, diarrhea, vomiting, nausea, neutropenia, fatigue, and hypokalemia [148]. These results suggest efficacy and safety of narsoplimab as treatment for HSCT-TMA.

In patients with HSCT-TMA, there is an inherent risk of infection due to intensive conditioning regimens and prolonged immunosuppression associated with the transplantation process. HSCT recipients should be given vaccinations according to published guidelines [149], although there is limited evidence regarding the efficacy of meningococcal vaccinations in HSCT patients receiving terminal complement inhibitors [150, 151]. Antibiotic prophylaxis should be considered for patients receiving C5 inhibitors despite vaccination status [151]. Following HSCT, and in those receiving complement inhibitors for HSCT-TMA, it is critical that patients are closely monitored for infection.

## Conclusions

The characterization of endothelial injury and complement activity in human diseases has improved our understanding of HSCT-TMA and other EIS. Genetic, histologic, and clinical evidence supports the “Three-Hit Hypothesis” for HSCT-TMA, demonstrating that pre-existing physiologic conditions as well as peri-transplant events and immunologic agents add to the risk of endothelial injury in patients undergoing HSCT.

Currently, there is limited knowledge of the natural history of HSCT-TMA in adult patients. A better understanding of the clinical course of HSCT-TMA in both adult and pediatric patients is needed to provide appropriate treatment. Diagnostic and prognostic markers will be important for distinguishing between patients who may benefit from supportive care versus anti-complement therapy.

Understanding the pathophysiology of complement activation in EIS, including activation of the lectin pathway, has provided an opportunity for evidence-based and mechanism-based, targeted therapy for HSCT-TMA. Preliminary single-arm clinical trials evaluating complement inhibitors for treatment of severe HSCT-TMA have provided promising results for this life-threatening condition. Lectin pathway inhibitors hold potential for treatment of HSCT-TMA.

## Data Availability

Not applicable.

## Abbreviations

ARDS: Acute respiratory distress syndrome
CH50: Classical pathway hemolytic assay
CL: Collectin
CLS: Capillary leak syndrome
CRP: C-reactive protein
DAH: Diffuse alveolar hemorrhage
DAMP: Damage-associated molecular pattern
EASIX: Endothelial Activation and Stress Index
EIS: Endothelial injury syndromes
ES: Engraftment syndrome
GVHD: Graft-versus-host disease
HSCT: Hematopoietic stem cell transplantation
HSCT-TMA: Hematopoietic stem cell transplantation-associated thrombotic microangiopathy
IgG4: Immunoglobulin gamma 4
IPS: Idiopathic pulmonary syndrome
LDH: Lactate dehydrogenase
MAC: Membrane attack complex
MAGIC: Mount Sinai Acute GVHD International Consortium
MASP: Mannan-binding lectin-associated serine protease
MBL: Mannose-binding lectin
mTOR: Mammalian target of rapamycin
PRM: Pattern-recognition molecule
REG3α: Regenerating islet-derived 3α
SARS-CoV-2: Severe acute respiratory syndrome coronavirus 2
SOS: Sinusoidal obstruction syndrome
ST2: Suppressor of tumorigenicity 2
TA-TMA: Transplant-associated thrombotic microangiopathy
TMA: Thrombotic microangiopathy
TNF-α: Tumor necrosis factor-α
TPE: Therapeutic plasma exchange
VOD: Veno-occlusive disease
